# Setting adequate wages for workers: Managers’ work experience, incentive scheme and gender matter

**DOI:** 10.1371/journal.pone.0271762

**Published:** 2022-08-17

**Authors:** David Huber, Leonie Kühl, Nora Szech

**Affiliations:** 1 Karlsruhe Institute of Technology, Chair of Political Economy, Karlsruhe, Germany; 2 CESifo, Munich, Germany; 3 Berlin Social Science Center (WZB), Berlin, Germany; Heidelberg University, GERMANY

## Abstract

Many societies report an increasingly divergent development of managers’ salaries compared to that of their workforce. Moreover, there is often a lack in diversity amongst managerial boards. We investigate the role of managers’ gender and incentive scheme on wages chosen for workers by conducting two experimental studies. The data reveal male managers respond in more self-oriented ways to their incentive scheme. Further, we find that experience with the workers’ task can increase appreciation of workers. Effects are strongest when the managers’ compensation scheme rules out self-orientation. Overall, female managers display more consistency in choosing adequate wages for workers, i.e. their choices are less affected by incentives. An increase in diversity may thus help reducing salary disparities and foster work atmosphere.

## 1 Introduction

“Surely no sensible person would say that management is not an art”, writes Henry M. Boettinger, director of corporate planning at AT&T in 1975 in Harvard Business Review [1]. More recently, however, it has been criticized that the training at business schools may foster ruthless behavior and greed in managers [2]. Selfishness in managers has also been addressed as a reason behind the VW emissions scandal in 2015 [3, 4]. Legislation has tried to curb selfish behavior in CEOs, e.g. by enforcing disclosure of CEO-workforce gaps in the US [5]. Nevertheless, there is still a vibrant public debate about setting rigid boundaries to CEO earnings [6–8]. There is evidence that managers with stronger prosocial preferences can have positive effects on both their workers and shareholders’ revenues [9]. Still, it has been argued that managers at the top hierarchy level, if having the opportunity, may try to maximize own salaries, and that employees may directly or indirectly suffer from this [10, 11].

Alongside this public discourse about ethics, greed and morality in business, the number of women in management positions is steadily growing, though overall numbers are still low in many sectors (compare e.g. the share of females on boards in OECD countries [12]). Many reasons and policy approaches for this have been discussed (see, e.g. [13–15]). Literature from economics and psychology suggests women may be more morally inclined than men. In survey studies, a gender effect does not always exist, yet when it does, it is typically in favor of women [16–19]. Thus, increasing gender diversity in management may bear potential for the well-being of dependent workers and work atmosphere. With an overall still moderate number of females in management positions, it is of course difficult to assess the influence of female leaders. Yet there is at least some evidence that female managers have a positive effect on work atmosphere and wages paid to employees [20, 21]. In these studies, however, the question of causality remains unanswered.

Therefore, in two gender-controlled experimental studies, we explore the influence of incentive schemes, manager’s gender and work experience on appreciation of workers, reflected in wages chosen. Subjects are randomized into the roles of managers or workers. While workers spend an hour constructing and deconstructing pens, an arguably tedious task, managers decide over the workers’ compensations. We vary managers’ personal incentives, notably, whether they can maximize their own earnings or not, as well as their experience with the workers’ task across treatments.

This results in the four following treatments. In *Baseline*, managers decide what they consider an adequate compensation for workers, up to a maximum amount. All money not awarded to the worker stays with the university and is used for further research projects. In the *Self* treatment, managers are faced with the same task but can keep all money not spent on their worker’s compensation for themselves. Two additional treatments, *ExpBaseline* and *ExpSelf*, are similar, respectively, except that managers acquire some work experience prior to their decisions on workers’ compensations. Managers construct and deconstruct one pen themselves before choosing an adequate compensation for the workers. Next to treatment effects, we focus on the interaction of treatment and manager’s gender.

Our main findings are as follows: Incentive schemes matter. Workers’ compensations in *Self* are lower than in *Baseline*. This happens even though compensation in *Baseline* is already moderate compared to the local minimum wage. Comparing *Baseline* to *Self*, female managers show a more consistent choice of compensations than their male counterparts, showing a smaller effect of the underlying incentives. Both of these results are replicated in the respective treatments with experience (*ExpBaseline* and *ExpSelf*).

Work experience significantly increases compensation overall. However, this increase is mainly driven by higher compensations paid in *ExpBaseline*, where high compensations for workers do not reduce managers’ earnings. In contrast, compensations in the respective *Self* treatments remain at a low level, no matter whether managers are more or less familiar with the workers’ task.

Additionally, we measure workers’ beliefs on adequate compensations for the task, as well as what they expect managers to choose in their treatment. Workers’ perceptions of an adequate compensation is stable across treatments. Anticipating the behavior of others, or own behavior in a different situation or emotional state, can be difficult (compare e.g. Van Boven and Loewenstein [22] and the references therein). Our data reveal that nevertheless, workers are aware that managers’ incentive schemes will impact their wage choices.

It has been argued that experience with the tasks workers have to do may help managers to create a more suitable work atmosphere [23, 24]. In the hotel and tourism sector, for example, it is widespread practice that an apprenticeship (that can also aim for higher management levels), starts with hands-on work as a dishwasher in the kitchen or as a concierge [25, 26]. In other industries, managers also work at lower levels to get a feel for the tasks and challenges first hand, including managers at Tesco [27] and Morrisons [28]. United Parcel Service highly values personal experience of leaders and managers with tasks like sorting packages or driving a package car. When hiring from within, such experience is a must. For newcomers, special hands-on trainings are in place [29]. Our results show, however, that the impact of experience is limited when it comes to a costly appreciation of workers.

This paper contributes to the literature on gender differences in ethical decision making. So far, most studies rely on questionnaires and hypothetical scenarios (see e.g. [18] for an overview), also when it comes to managers [21]. This line of research suggests females behave in more ethical ways. In the economic experimental literature, a survey study by Croson and Gneezy [30] reports similar findings when it comes to altruistic behavior. Further, males lie more often to secure a monetary gain than females [31]. This is also observed in group behavior [32] and in competition [33]. However, males prove very generous and moral if it does not lead to economic disadvantage for themselves [34]. Our results are in line with this.

To the best of our knowledge, no economic study so far has considered the impact of own work experience on the appreciation for others carrying out similar work. The data shows that such experience in managers can increase wages chosen for workers. Effects are strongest when higher wages don’t have a negative effect on the managers’ payoff. In contrast, especially male managers stay rather self-oriented when they weigh workers’ wages against their own. Experience with the workers’ task does not reduce this self-orientation.

One may argue that also in dictator games, many subjects give considerably less to the other party than what they take for themselves (see Engel [35] for a survey). In fact, dictators who give about 30 percent of the total are often classified as “rather altruistic” [36]. In these games, the situation is typically symmetric across dictator and recipient, only decision power is asymmetric. In contrast, in our setting, workers have to carry out a tedious task that managers know takes about an hour to complete, while managers only decide about wages and can leave the premises of the study much faster. Nevertheless, on average, managers cash in a lot more of the overall “pie”, if they can.

Possible reasons for female under-representation in higher management positions are manifold. It has been argued that males may compete too much, while females may compete too little [37–39] (Research on children reveals this may also depend on the task [40, 41]). This difference in willingness to compete is reported to be driven by, among other factors, differing distributional preferences [42]. Other reasons include taste-based discrimination [43], statistical discrimination [44, 45], and discrimination from biased beliefs [46–49]. Furthermore, women are reported to be less self-promoting which stems from a lesser subjective evaluation of their own performance [50]. It has also been documented that competitive environments can enhance sabotage [51, 52], and that females may engage less in sabotaging others than males [53, 54]. No matter why less females may end up in top management, our studies suggest that appreciation of workers may suffer from a lack of diversity in management positions.

## 2 Experimental design and hypotheses

**Fig 1:**
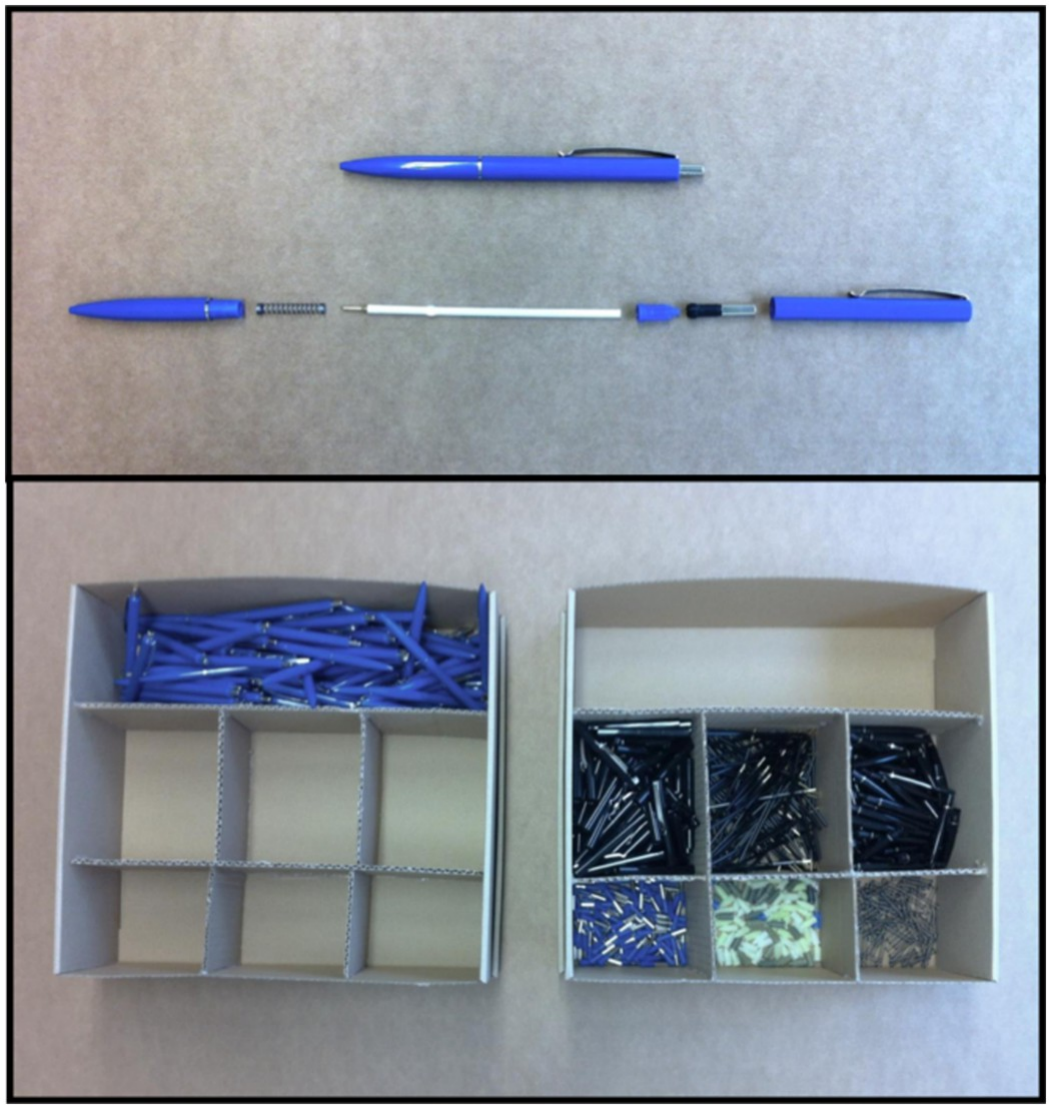
Working materials as depicted in managers’ instructions. The first photo shows one assembled and one disassembled pen. The second photo shows the sorting boxes workers work with during the task.

In our experiments, subjects are randomized into the roles of managers and workers. In all treatments, managers decide independently over the compensation (or wage) of one worker. Managers know that their worker has to work on a tedious task: the complete assembly of 100 pens and the complete disassembly of another 100 pens. The working task is described in the instructions (Instructions are provided in the S1 Appendix, section A.4) and illustrated with two pictures of the materials workers use (pens and sorting boxes, compare Fig 1). Managers further know that the completion of the task requires about one hour of working time and that workers will be paid only after completion of the task. Also, the manager’s payoff does not hinge on the worker actually finishing the task. In all treatments, managers are asked to decide over what they consider the *adequate* compensation for their worker. Compensation can be set in 30 cents steps ranging from 0 to 21 euro.

We run two studies. In the first study, we vary gender of managers and incentive structure. Every manager makes exactly one decision, namely setting the wage for *one* worker. In the *Baseline* treatment, managers decide over an adequate compensation facing the trade-off towards a common public interest (funding non-related research). They are asked to allocate the total amount of 21 euro according to what they consider the adequate compensation for the workers, knowing that the rest of the money goes to the university’s research budget and will be used for other research purposes.

In the *Self* treatment, in contrast, managers weigh their own interest against that of a worker. They decide how much the worker should receive as an adequate compensation, knowing they will keep the rest to themselves. Here, managers’ self-interest should play an important role and influence how compensations for workers are chosen. Depending on company size, owners acting as mangers may face a trade-off of this kind. In larger companies, managers may rather have to compare different important interests. This may fit better to the *Baseline* treatment.

Our design removes all monetary incentives for managers to motivate their workers with a high compensation. Thus, we can focus on their distributional preferences. Managers know that the situation is very asymmetric: Workers have to work on the task for about an hour, whereas managers do not face the real-effort task. Accordingly, managers have a much lower time investment.

Next to treatment effects, we focus on the role of gender in managers. Therefore, we run a gender-controlled study. We invited similar numbers of males and females to each session. Interaction between an experimenter, who was blind about the hypotheses of the study, and participants was reduced to an absolute minimum. We further kept the gender of the experimenter constant over all sessions.

As we observe rather low wages being chosen in our first study, especially by male managers, we conduct a second study to explore a channel which might raise workers’ wages. We investigate if managers choose higher compensations when they know the workers’ task from own experience. Therefore, in the second study, managers gain personal experience with the working task in two new treatments: *ExpBaseline* and *ExpSelf*. Managers completely assemble and disassemble one pen each, before they determine what they consider an adequate compensation for workers. Incentives are identical to the corresponding prior treatments from the main study, *Baseline* and *Self*, respectively. We further conduct an additional control treatment to make sure that the sole presence of working materials does not cause a change in perception of the working task. For details and results, see S1 Appendix section A.2. Similar to the first study, we conduct the second study in a gender-controlled way.

### 2.1 Details of the studies

A total of 500 participants take part. Participants are recruited from a mixed student subject pool using ORSEE [55] and HROOT [56] in a gender-controlled manner. They are randomized into the roles of worker or manager. For further details, see S1 Appendix section A.1.

All participants receive a show-up fee of 5 euro. At the beginning of each session, workers receive information about their working task and the decision their respective manager faces. They are informed that they get to know and receive their wage after they complete the working task which lasts about one hour. Before they start their work, they fill in a short, mostly socio-demographic questionnaire. In addition, they indicate what they consider an adequate compensation for their work as well as what actual compensation they anticipate from their manager. Depending on managers’ decisions, workers receive between 0 and 21 euro for their work. Workers stay about 75 minutes in the lab.

Managers do not have to complete the workers’ task. After they decide upon workers’ wages, they are asked to complete a survey on norms, personal characteristics and demographic background for an extra compensation of 5 euro. All managers choose to take part in this. In *Baseline*, managers thus receive 10 euro in total. In the *Self* treatments, they receive 10 euro minimum and 31 euro maximum, depending on the compensation they choose for their workers. Managers stay about 45 minutes in the lab.

All participants complete their sessions except for one female worker who decides to leave the study after reading the instructions. Another female worker is retroactively matched with the replaced worker’s manager (Since all participants were already in their cubicles, the departure of this participant went unnoticed and could not have impacted any decisions by other participants in the session).

### 2.2 Hypotheses

Prosocial preferences matter, yet typically, they matter less than selfish interests, compare, e.g., Engel [35] This motivates our first preliminary hypothesis.

**Hypothesis 1**: *Managers choose higher wages for workers in* Baseline *than in* Self.

Building on this precondition, we hypothesize that this difference between treatments is less pronounced in female managers. There is a rich literature from business ethics showing that females tend to behave in more moral and less corrupted ways (Compare surveys by O’Fallon and Butterfield [18] and Craft [57] for an overview based mainly on hypothetical scenarios or questionnaires; see Deckers et al. [17] for evidence from real, incentivized decisions). Therefore, female managers may have a higher capacity to resist temptations in the *Self* treatments to keep more money for themselves compared to male managers. Furthermore, evidence exists that men are more generous than women only if said generosity comes cheap [34]. Therefore, for our main hypothesis, we expect female managers may to display a smaller difference between chosen workers’ wages across *Self* and *Baseline* treatments.

**Hypothesis 2**: *The difference between wages for workers in* Baseline *and* Self *is larger for male than female managers. That is, female managers are less influenced by the incentive scheme they are in*.

After analyzing the results of our first study, we set out to explore a mechanism to combat selfish interests in managers. Own work experience has been discussed to increase valuation for a work others carry out and empathy with those who conduct it (compare e.g. Louis [23]). Many companies expect their future managers to first gain a hands-on impression of the work employees on lower hierarchy levels carry out [27, 28]. This motivates our treatments of the second study and leads to the following hypothesis.

**Hypothesis 3**: *First-hand experience with the workers’ task increases wages managers choose for workers*.

We also elicit the beliefs of the workers and their perspective on an appropriate compensation for their work, which should be independent of their managers’ incentives. However, we theorize that workers are able to anticipate how these incentives influence the chosen wages.

**Hypothesis 4**:

***a***) *The wage a worker considers appropriate for their work does not hinge on the incentive scheme the managers face*.

***b***) *Workers predict managers’ incentive schemes to affect the wages they receive for their work*.

## 3 Results

In section 3.1, we present the results of our first study which focuses on gender and incentive scheme of managers. Results of the second study introducing experience with the working task are provided in section 3.2. Section 3.3 presents our findings about workers’ beliefs and expectations. Further results as well as a consolidation of our findings can be found in S1 Appendix section A.3.

### 3.1 Incentive scheme and gender

The focus of our first study is the impact of managers’ gender, incentive scheme and the interaction of the two on wages for workers. Firstly, we find that wages chosen by managers react to incentive schemes, i.e. workers’ compensations in *Self* are lower than in *Baseline*.

In line with Hypothesis 1, the different incentive schemes have a significant effect on workers’ wages. While managers in *Baseline* choose 10.29 euro on average as an appropriate wage for workers, managers in *Self* paid only 8.06 euro (p = 0.000***, two-sided t-test. Please note that we report results of t-tests as this is a rather robust test even if some assumptions are not perfectly met—for instance, normal distribution and continuity of data [58], compare S1 Appendix section A.3.2).

Managers in *Baseline* know that money not spent on the worker is used for a public good, i.e. future university research. Participants seem to have seriously considered this trade-off as only one manager chooses the maximum compensation of 21 euro for workers. Further, one manager (in the *Self* treatment) chooses to award 0 as workers’ wage. Our results remain robust if we exclude one or both of these border cases. Looking at the level of wages, compensation is generally rather low with a substantial part of managers rewarding less than the legal minimum wage at the time. 21.1% of managers in *Baseline* and 42.4% in *Self* choose less than 8.50 euro which is the hourly minimum wage when the study takes place.

Managers spend 45 minutes on average in the lab while workers stay there for 75 minutes. In the *Self* treatment, managers take 35.88 euro as an hourly payoff on average. Thereby, they earn about 3.5 times as much as workers. This discrepancy arises even though workers arguably have a tedious task to carry out.

At first glance, it might look like there is no effect of gender on wages chosen. Pooling data over both main treatments, female and male managers pay 8.97 euro and 9.28 euro on average, respectively. This difference is not statistically significant at any conventional level (p = 0.61, two-sided t-test). Similarly, comparing the two treatments separately, differences are relatively small. Male managers are a bit more generous in *Baseline* (11.10 euro vs. 9.44 euro, p = 0.076*, two-sided t-test) while female managers award insignificantly more in *Self* (7.59 vs. 8.54, p = 0.16, two sided t-test). When we take a closer look at the data, however, we can confirm that female managers show less variation across the two treatments.

**Fig 2:**
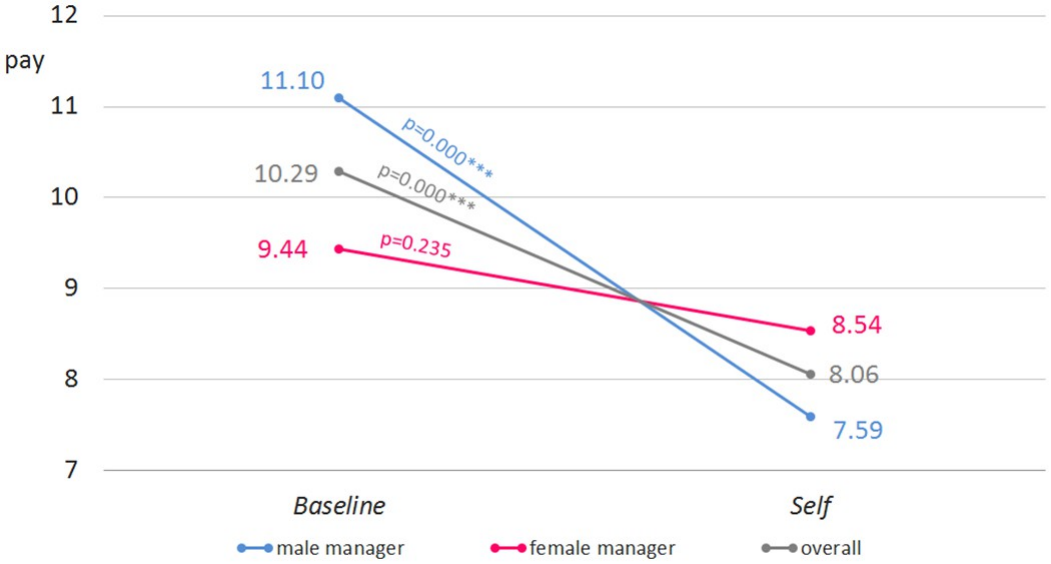
Study 1 by gender. Pay chosen by manager’s gender (in euro) in *Baseline* vs. *Self* (male vs. female manager, diff-in-diff, p = 0.027**, one-sided t-test, n = 114).

We hypothesized that male and female managers react differently to the different incentives. In line with Hypothesis 2, the data reveal this is the case. While the workers’ wages chosen by male managers amount to 11.10 euro in *Baseline*, they drop to 7.59 euro in *Self* (p = 0.000***, two-sided t-test). Thus, the data show a clear causal effect of the incentive scheme on male managers. Female managers choose 9.44 euro in *Baseline* compared to 8.54 euro in *Self* (p = 0.235, compare Fig 2). The respective difference in difference measure is significant and therefore supports our main hypothesis (p = 0.027**).

Our post-decision survey of managers revealed that their chosen wages are in line with their evaluation of the workers’ task as well as their opinion on minimum wage. Note, however, that this survey was not incentivized, so subject might have used it to explain or justify their decision retroactively. We elicit managers’ evaluation of the task in 5 different dimensions (with 7-point Likert-scales each): how honorable and how hard the task is considered as well as how demanding in terms of special abilities, technical skill and spatial sense. We also elicit managers’ approval of a legal minimum wage with a 7-point Likert-scale. We find strong positive correlations between wages payed and both a higher evaluation of the task as well as positive opinions on minimum wage (OLS regression, p = 0.021** and p = 0.013**, n = 113, for more details, see S1 Appendix section A.3.1).

### 3.2 Introducing experience

In our second study, we find that personal experience of managers can overall increase workers’ wages. Yet, its effect is mostly limited to the treatment in which self-interest was absent, i.e. *ExpBaseline*. As the evaluation of the work (as captured by the manager survey) is not affected by experience, we conclude that the increase in what is considered an adequate compensation is due to a change in appreciation of the worker. Again, and in line with our first study, female managers choose wages more consistently. Behavior of male managers hinges on the underlying incentive scheme more strongly.

**Fig 3:**
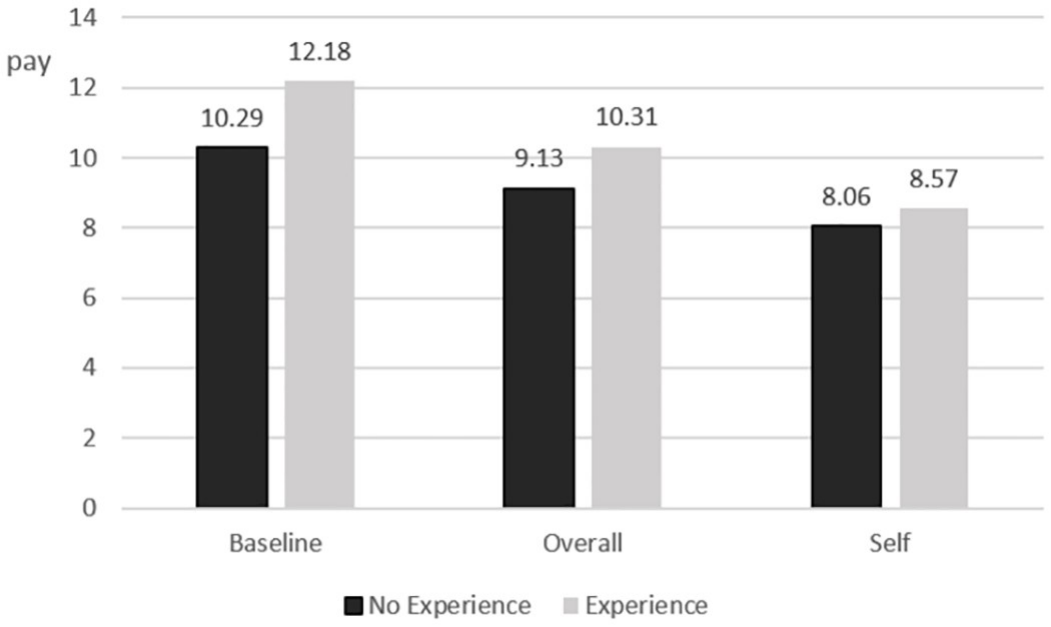
Adequate pay, incentives and experience. Chosen wage (in euro) in *No Experience* vs. *Experience*.

Wages in *ExpBaseline* and *ExpSelf* average 10.31 euro compared to 9.13 euro in the corresponding treatments without experience, i.e. *Baseline* and *Self* (p = 0.018**, two-sided t-test). This is in line with Hypothesis 3. However, this increase from work experience is almost exclusively driven by higher compensations in *ExpBaseline* compared to *Baseline* (12.18 vs. 10.29, p = 0.009***). Wages in the treatments where self-interest plays a role remain virtually unchanged and are not significantly different at any conventional level (8.57 in *ExpSelf* vs. 8.06 in *Self*, compare Fig 3). Thus, personal experience with the worker’s task seems to be able to increase the appreciation for the worker but there is no evidence that it can counteract effects of selfishness in managers.

Pooling both experience treatments shows no effect of gender on compensations (10.58 euro for female vs. 10.05 euro for male managers, p = 0.498). The same is true for *ExpBaseline* (11.68 for female managers vs. 12.70 for male, p = 0.355). In *ExpSelf* male managers choose significantly less this time (9.52 vs. 7.66, p = 0.041**).

**Fig 4:**
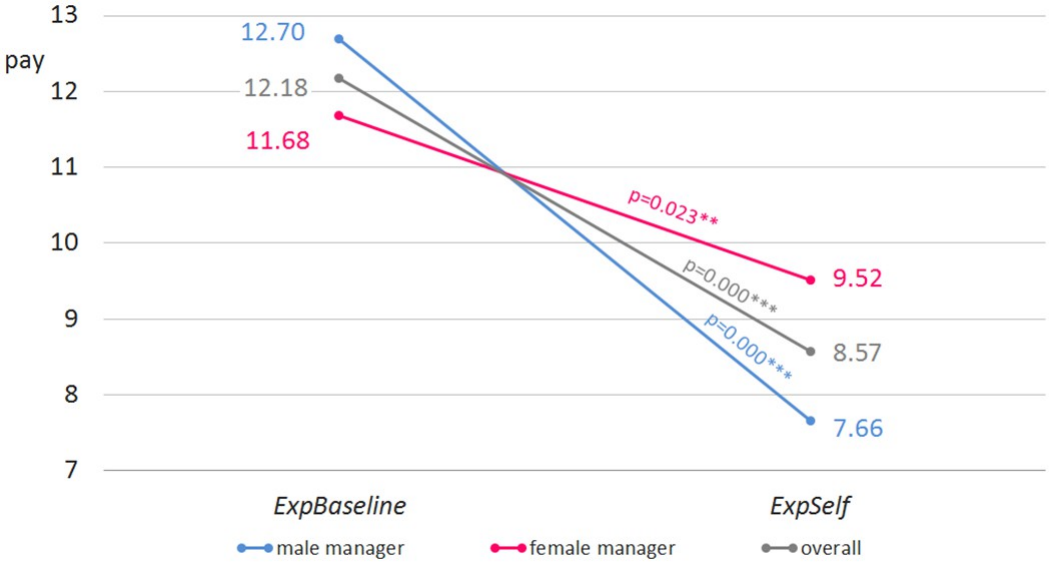
Study 2 by gender. Pay (in euro) in *ExpBaseline* vs. *ExpSelf* (male vs. female manager, diff-in-diff, p = 0.041**, n = 114).

Again we find the hypothesized effects when looking at the interaction of gender and incentives, with male managers showing stronger treatment differences. Male managers in *ExpBaseline* choose 12.70 euro while those in *ExpSelf* choose 7.66, a highly significant reduction of 40% (p = 0.000***). Compensations chosen by female managers also drop significantly (11.68 euro vs. 9.52 euro, p = 0.023**). Yet, this reduction is lower than for male managers, which is confirmed by a significant difference-in-difference measure (p = 0.041**) (compare Fig 4). Again, female managers decide more consistently across incentive schemes, replicating our main result from the first study.

The managers’ survey showed no change in the evaluation of the workers’ task due to personal experience. So familiarity with the task does not affect how challenging or demeaning the task is perceived by managers. Yet, at least in *ExpBaseline*, managers are willing to pay a significantly higher amount to their workers. This suggests that the higher wage may be caused by a higher appreciation of the worker rather than a different relation with the task.

### 3.3 Workers’ beliefs and expectations

In this section, we present our results of the elicitation of expectations and beliefs of the workers. Workers answered these questions *before* working on their task. We find that worker’s beliefs on what is an adequate compensation does not depend on the incentive scheme the manager faces and are generally higher than wages the managers actually choose.

**Fig 5:**
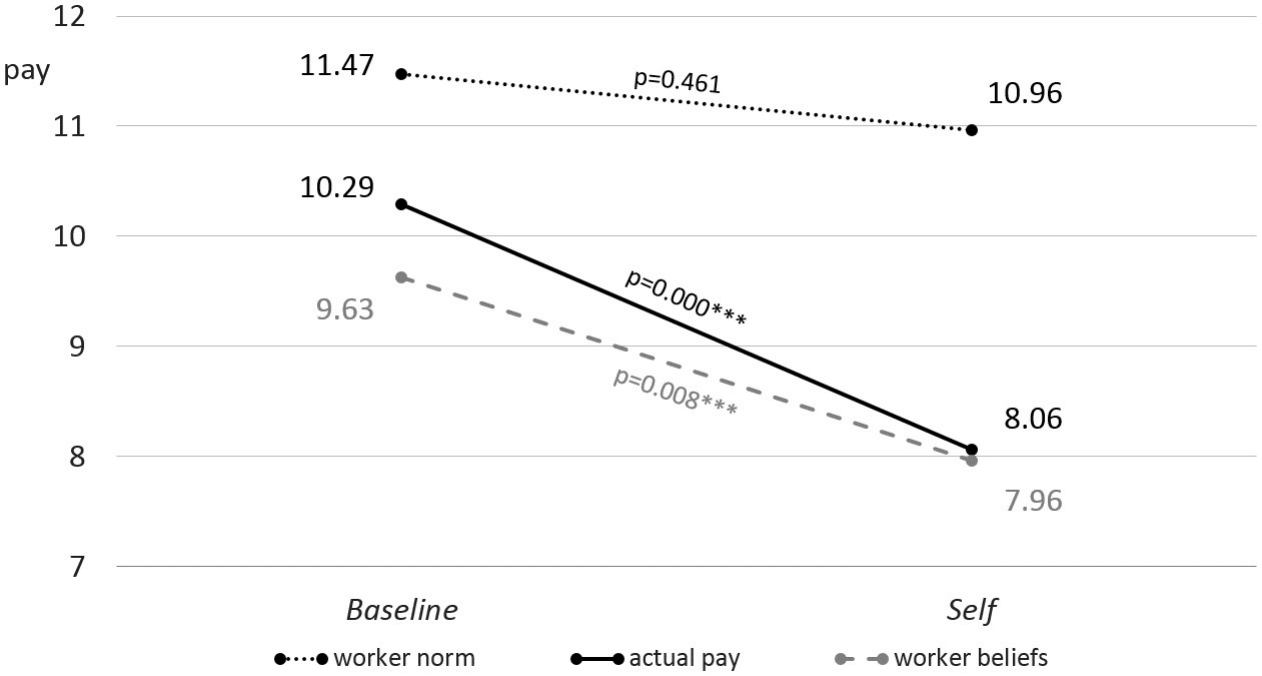
Worker norm and beliefs (Study 1). The worker norm differs significantly from actual pay in *Baseline* and *Self* (11.47 euro vs. 10.29 euro and 10.96 euro vs. 8.06 euro, two-sided t-test, p = 0.090* and p = 0.000***, n = 110 and n = 116, respectively). Worker beliefs, however, do not differ significantly (p = 0.347 and p = 0.830, respectively).

Workers perception of what is an adequate compensation of the task is consistent between treatments (11.47 in *Baseline* vs. 10.96 in *Self*, p = 0.461), confirming Hypothesis 4a. This is more than the wages that were actually paid in both treatments, respectively (11.47 euro vs. 10.29 euro, p = 0.090* and 10.96 vs. 8.06, p = 0.000***). Workers predict managers’ decisions quite well. The data display no significant differences between workers’ expectations and compensations chosen by the managers (10.29 euro vs. 9.63 euro in *Baseline* and 8.06 vs. 7.96 in *Self*, also compare Fig 5). This suggests that workers are able to accurately estimate the effect of the incentive scheme on managers’ decisions.

## 4 Discussion

This study provides evidence that incentive scheme, gender and experience matter for what managers choose as an adequate wage for workers. In the following section, we discuss some limitations and special characteristics of this study.

Our experiment represents a highly simplified employer-employee relationship between a manager and a worker. We explicitly and deliberately focus on the wage a manager chooses for workers for the completion of a task. The design leaves no room for gift-exchange or reciprocity (compare e.g. [59–61]) or negotiation (compare e.g. [62–64]). Of course, this abstracts from many real-world scenarios in which decisions might be influenced by those mechanisms. However, by doing so, we ensure that managers are not affected by other considerations than those we aimed for: the wage they deem appropriate for completing a specific working task.

In real world settings, the wage for work is often tied to the value of the work, for example the market price for a provided service or a product. In that sense, the value of the workers task in our experiments is arguably zero and the wages chosen by managers can be seen as a lower boundary of their willingness to pay (or award) for an hour’s work. But while this design choice very likely adds to the overall low level of wages we observe, it also lets us control for different values managers might attribute to the task, which could otherwise distort our data.

The design of our study borrows some elements of a standard dictator game. For a review and meta study on dictator games including findings on gender differences and framing, see Engel [35]. Specifically in our *Self* treatment, managers weigh their own benefit against money another participant of the study receives. However, standard dictator games cannot directly relate to behavior in pay determination as they usually abstract from labor situations and effort using simple endowments entitled to the dictator for no specific reason. This means that, first, social obligations of dictators (i.e. managers in our study) resulting from labor are basically absent (compare e.g. Blau [65]). Second, giving in dictator games can be seen as a measure for altruism (compare e.g. Eckel and Grossman [66]), not a measure for valuation of work as it is in our study. Of course, there exist studies, that use real effort tasks in dictator games instead of endowments [67, 68] as well as social framings (compare e.g. Dreber et al. [69] and the references therein). We feel assured by their results that a real effort task and the design of our study lead to a feeling of social responsibility towards workers (compare e.g. Handgraaf et al. [70]) that would not apply in a standard dictator game. However, additionally to the differences mentioned above, the goal and scope of these studies is a completely different one: they investigate how real-effort tasks change giving/taking amounts compared to games where effort is absent. Our study, however, uses a fixed level of effort and focuses on how a change in trade-offs, gender and own work experience causally affects the valuation of work done by others. Our managers have to decide what they consider an adequate pay for workers who have to work on a tedious task for about an hour—which they themselves do not have to complete. Furthermore, we compare this to a baseline treatment in which this decision is independent from own profits of managers to find out how valuation for work is affected by monetary incentives.

As we use a student sample of the Karlsruhe Institute of Technology, our results are, of course, not representative for the general population. However, this specific sample might be an especially interesting one as the majority of Germany’s CEOs are former students of this university [71]. Thus, we conduct this experiment with a sample that is not representative but potentially even more relevant for the considered context.

Debates on the social acceptability and adequacy of (extreme) wages are a recurring and old phenomenon [72–74]. Fairness concerns in labor markets and wage negotiations have already been addressed by Slichter [75] and Hicks [76]. In our experiments, we abstract from market forces and focus on the valuation for work done by others. Following the origins of the standard neoclassical model, wage determination is a matter of supply, demand and productivity. However, it has been argued that it is not markets alone that determine wages but that labor markets are more or less imperfect which allows for several other mechanisms to play their part in determining wages [77]. Furthermore, social economics suggests a different approach: wages should not be considered a price but should adequately reflect workers’ needs [78]. Williams [79] stresses that the two opposing views of wages as a price on free markets vs. wages as the result of social and moral consideration (and thus being part of a state’s responsibility) remain hard to align. Including market forces into our approach in future research could potentially help to understand how these two aspects interact.

## 5 Conclusion

In this paper, we conducted two studies to investigate how managers choose wages for workers. Wages chosen hinge on the manager’s gender, whether managers have some experience with the worker’s task, and the incentive scheme the manager is in. As expected, when managers can profit from paying lower wages, they generally do so. Yet, male managers react stronger to incentives schemes than females. Female managers exhibit a more consistent behavior which seems to be more robust to the opportunity to be selfish. These results may have important implications for organizations and work cultures and can add to the current discussion on gender compositions of board and upper corporate hierarchies.

First-hand experience with the worker’s task can increase the wages managers chose for workers. Yet, this effect is mostly limited to incentive schemes in which managers have no self-oriented reason to set low wages for workers. Therefore, first-hand experience with the workers’ task may soften disparities across workers and managers only in cases in which disparities were not so large to begin with.

What workers consider an appropriate wage for their task does not hinge on the managers’ incentive scheme. Yet workers predict an influence of the managers’ incentive scheme on the wages they will receive. This shows there is some awareness in workers, that may well contribute to social debate.

## Supporting information

S1 Appendix (PDF)
