# Supplementary material for: Setting adequate wages for workers: Managers’ work experience, incentive scheme and gender matter
Source: PLoS One. 2022 Aug 17;17(8):e0271762. doi: 10.1371/journal.pone.0271762 (PMC9385021; doi:10.1371/journal.pone.0271762)
Supplement: S1 Appendix — (PDF) [file pone.0271762.s001.pdf]

A Appendix

A.1 Study Details

Our double-blind and gender-controlled experiments were conducted between March and September 2015 (*Baseline*, *Self*) and between September and November 2016 (*ExpBaseline*, *ExpSelf*). There was a total of 500 participants, 250 managers and 250 workers (Compare Figure 1 which excludes 44 participants of the control treatment *BoxBaseline* which is described below). We used the software ORSEE (Greiner, 2015) and HROOT (Bock et al., 2014) to recruit from a mixed student subject pool. The data was elicited at the KD2 Lab of the Karlsruhe Institute of Technology, a German university. To be eligible to participate in economic experiments at the KD2 Lab, potential participants must register to be added to the subject pool. The registration serves as a declaration of intent to participate in experiments. Additionally, specifically for our two studies, all participants signed a contractor's declaration before being admitted into the lab. It has been carefully ensured and stressed before and during the experiment that answers would be handled confidentially and that managers make their decision under complete anonymity. There was no contact between managers and workers at any time.

A.2 Control treatment: *BoxBaseline*

In *ExpBaseline* and *ExpSelf*, managers not only gain experience with the working task but also with the materials (i.e. the boxes and pens workers use). To make sure the sole presence of these materials is not responsible for a change in the perception of the work, we conduct an additional control treatment: *BoxBaseline*. In this treatment, managers know the task not only from a description and pictures, they are provided with the actual working materials (cardboard boxes and pens), all else is held constant to *Baseline*. *BoxBaseline* should be considered an additional control treatment. The additional data from *BoxBaseline* is only included in further robustness checks in section A.3.1. We do not find a significant difference in pay between *Baseline* and *BoxBaseline* (two-sided t-test, p=0.8630,

		trade-off	
		adequate pay vs. money for research	adequate pay vs. own payoff
level of experience	no experience, only description and photos from task	*Baseline* n=110	*Self* n=118
	with experience, task is tested with working materials	*ExpBaseline* n=110	*ExpSelf* n=118

Figure 1: Distribution of participants across treatments

n=77) or the evaluation of the working task (two-sided t-test, p=0.6099, n=77). We thus conclude that changes in *ExpBaselie* and *ExpSelf* do not originate from the presence of the materials provided but from the experience with the task.

A.3 Additional Findings

In the following sections of the appendix, we provide further findings of our experiments. First, we provide a comprehensive analysis of our findings as well as a robustness check in section A.3.1. Furthermore, we report the effects of worker gender in section A.3.2.

A.3.1 Consolidation of Main Results

In the following section, we analyze the results from both experiments in a comprehensive manner. For that, we include both, the treatments with and without personal experience with the working task in our considerations. If suitable, we may also include our data from the *BoxBaseline* treatment. We find that our treatment effects are robust for a variety of controls. We also consolidate the findings on consistency and find the patterns repeated. At last, we discuss the

characteristics and distribution of our data.

Interaction effects of gender and incentives are robust. When we compare the diff-in-diff measures of the treatments with and without experience, we find that in both cases, females decide more consistently across contexts. To further check our findings for robustness, we pool the data from all treatments[1] and conduct an OLS regression (compare table 1). We can confirm that gender of the manager, incentive scheme and the interaction of those two matter in decisions over adequate compensation (see column (1) in table 1). Including not only a control variable for experience, but also measurements for the task evaluation, the opinion on minimum wages and the university entrance degree (Abitur), we can, again, substantiate robustness of our findings (see column (2) in table 1). Furthermore, the regression shows that managers who evaluate the task as more challenging, demanding and worthy, are more likely to set higher pay as an adequate compensation. A positive opinion on minimum wages by law and own personal experience with the working task in our experiments is also correlated with a higher valuation for the work. We also include a control variable for the university entrance degree (Abitur) as a proxy for cognitive ability and find that managers without a university entrance degree tend to consider higher pay adequate. Surprisingly, we do neither find any effects of the Big-5 personality measures (Allport and Odbert, 1936) nor a correlation between a score for Machiavellianism (Christie and Geis, 1970) and the behavior of managers. We do find a correlation between participants' subject of study and the pay determined: students of business and economic sciences determine significantly lower amounts as an adequate pay (10.32 euro for other subjects vs. 9.11 euro for business and economic sciences, two-sided t-test, p=0.011**, n=250). Wang et al. (2011) addressed education in economics as a driver for selfish behavior and a more positive attitude towards greed. However, some bias and demand effects, for instance with regard to prior lessons on profit maximization, can not be ruled out in our data.

[1] As we do not find a significant difference between *Baseline* and *BoxBaseline*, we also include data from *BoxBaseline* in this regression.

	(1)	(2)
Self	-1.410^{**}	-1.174^{**}
	(0.623)	(0.591)
Male	1.218^{**}	1.609^{***}
	(0.602)	(0.572)
Self*Male	-2.624^{***}	-2.801^{***}
	(0.877)	(0.827)
Task Evaluation		0.159^{***}
		(0.045)
Opinion on Minimum Wage		0.322^{***}
		(0.118)
Education		-1.944^{*}
		(1.017)
Experience		1.299^{***}
		(0.419)
Constant	10.441^{***}	7.775^{***}
	(0.426)	(1.349)

Table 1: OLS Regression. The table shows OLS regression coefficients (wage as dependent variable, pooled data, n=250) standard errors in brackets. $***/**/*$ indicate significance on a 1-/ 5-/ 10- percent level respectively.

Looking at the distribution of chosen wages (compare figure 2 and figure 3), we find that the most common pay is 10.50 euro (12.8%) which equals a 50/50 split of the maximum amount between worker and third party or manager, respectively. Inequity aversion according to Fehr and Schmidt (1999) could be one potential explanation for this finding. We furthermore find accumulations at salient points like round numbers (6, 9, 12, 15 euro) and at 8.7 euro which is very close to the current minimum wage in Germany of 8.84 euro (BMAS Bundesamt für Arbeit und Soziales, 2017). Compare e.g. effects of salience or anchoring (Einhorn and Hogarth, 1981; Kahneman, 2003) – specifically, anchoring effects of minimum wages (Fehr et al., 2009) – and relative pay in perceptions of pay fairness (Scarpello and Carraher, 2008), for a potential explanation of these findings.

Our data slightly deviates from a normal distribution (according to a Shapiro-Wilk test, a Kolmogorov-Smirnov test and our interpretation of the plot in figure 3). As mentioned above, we mostly report results of t-tests which is a rather robust test regarding violations of assumptions – like deviations from normal distribution for the case of our data (Sawilowsky and Blair, 1992). Please note that our treatment effects are robust for using Wilcoxon rank-sum (Mann-Whitney) tests or Kolmogorov-Smirnov tests instead.

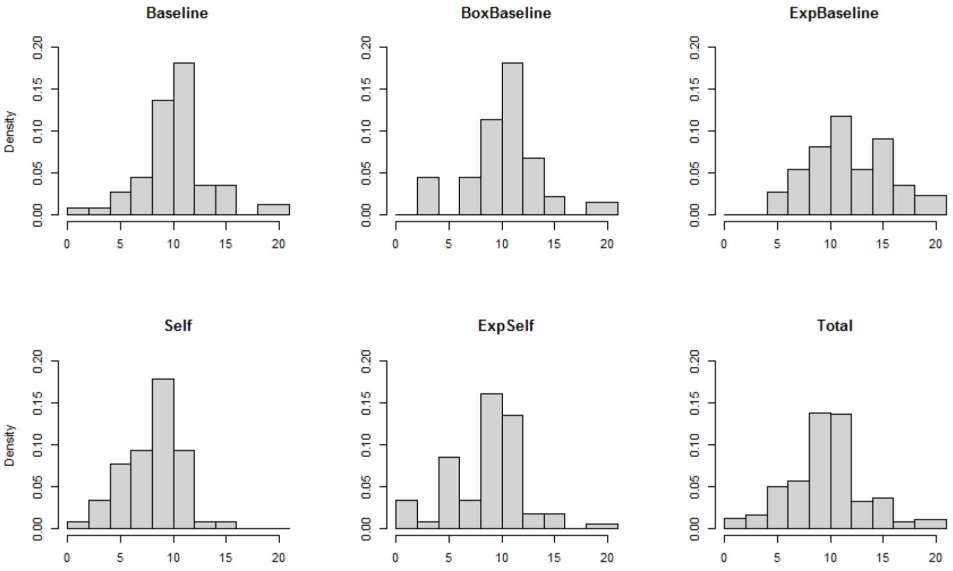

Figure 2: Histograms of pay. This figure illustrates (in a histogram for each treatment and in total) how pay is distributed between 0 and 21 euro.

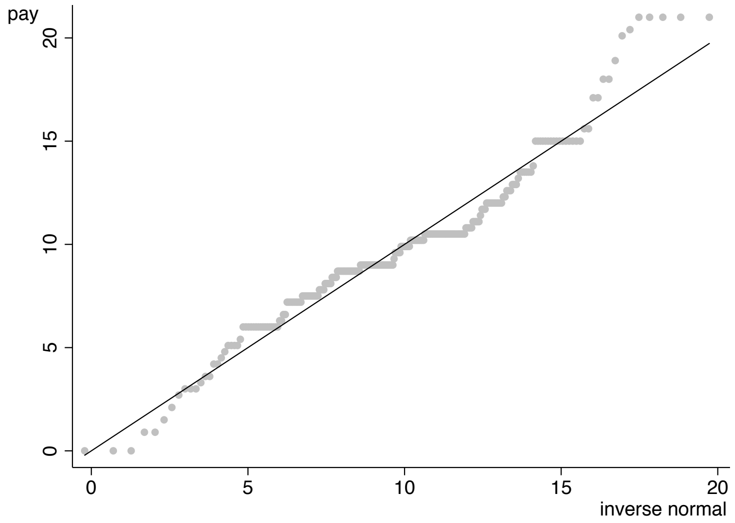

Figure 3: Distribution of pay. This figure illustrates the distribution of pay in comparison to a normal distribution (solid line).

A.3.2 Effects of Worker Gender

In the following section, we provide further findings on the effects of worker gender. In our double-blind and gender-controlled experiment, we also systematically vary the gender of workers to get some first insights whether worker gender matters. Overall, we do not find a significant difference in pay between female and male workers.

In *Baseline* and *ExpBaseline*, we do not find a difference in pay due to workers' gender overall (11.24 euro for male vs. 11.23 euro for female workers, data of *Baseline* and *ExpBaseline* pooled, two-sided t-test, p=0.994, n=110) or in the diff-in-diff measure (male vs. female, diff-in-diff, p=0.763, n=110, compare figure 4). Thus, our data shows no effect of workers' gender or the interaction of workers'- and managers' gender on the wages chosen in *Baseline* or *ExpBaseline*.

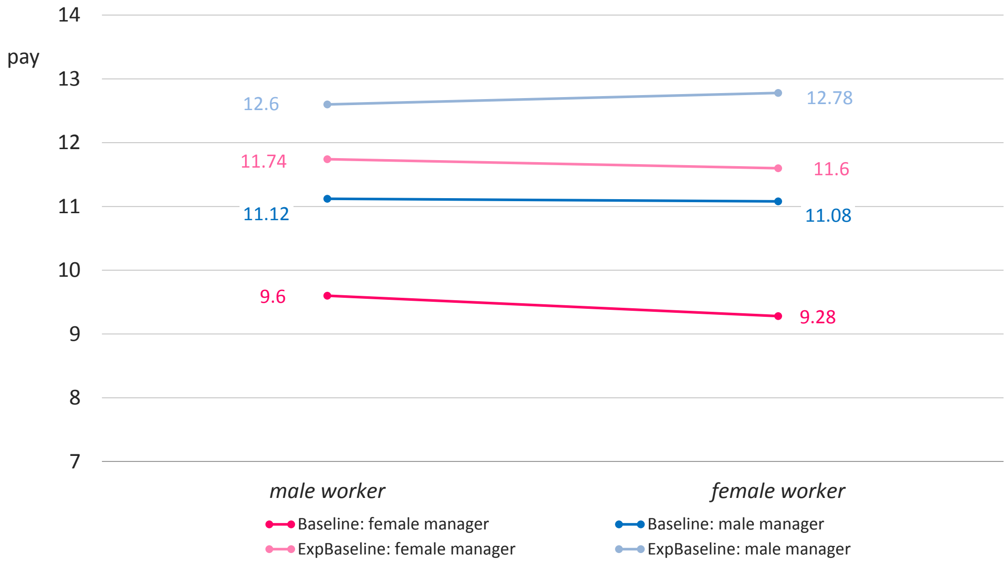

Figure 4: Interaction effect of manager gender and worker gender for *Baseline* trade-offs. Interaction effect of manager gender and worker gender in *Baseline* and *ExpBaseline* (male vs. female manager, diff-in-diff, p=0.763, n=110).

In the *Self* trade-offs, we find suggestive evidence that the gender of a worker affects pay. Again, we do not find an overall difference in pay for female vs. male

workers in *Self* and *ExpSelf* (8.36 euro for male vs. 8.27 euro for female workers, two-sided t-test, p=0.876, n=118). We find in our data that for contexts in which self-interest plays a role, male and female mangers both seem to slightly favor workers of their own gender (pooled data of *Self* and *ExpSelf*, diff-in-diff male vs. female managers, p=0.072*, n=118, compare figure 5). As this result was not hypothesised and all four pairwise differences (male vs. female worker, male or female managers, with or without experience) are not statistically significant, further investigation is necessary for confirmation.

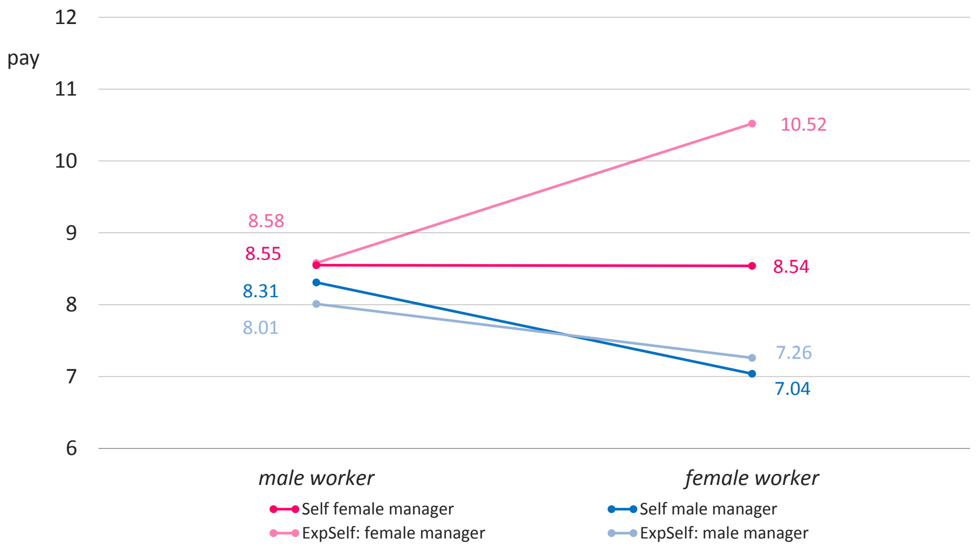

Figure 5: Interaction effect of manager gender and worker gender for *Self* trade-offs. Interaction effect of manager gender and worker gender in *Self* and *ExpSelf* (male vs. female manager, diff-in-diff, p=0.072*, n=118).

In psychology, but also other related fields, gender differences in compassion and empathy have been widely discussed (compare e.g. Eisenberg and Lennon (1983); Goetz et al. (2010); Rueckert et al. (2011)). Although evidence is somewhat mixed, females tend to be considered more compassionate and empathic (compare e.g. Schulte-Rüther et al. (2008)). Thus, one potential explanation for the gender difference we observe could be that female managers are simply more empathic and compassionate when they experience the task prior to the decision.

Considering the additional difference between male and female workers, a potential explanation could be some sort of in-group bias originating from a feeling of belonging to a group of certain gender (Charness and Rustichini, 2011).[2] Also, gender specific role perceptions or even stereotypes might suggest that female workers are more in need of support than males (who could be stereotypically seen as stronger and more resilient (Eagly, 2013; Diekman and Clark, 2015)). Maybe, female managers develop special commiseration towards female workers after having experienced the task themselves. Of course, further research would be needed to clarify the exact mechanisms at work.

Auspurg et al. (2017) have argued that females accept disadvantages in pay. Please note that we do not find an overall disadvantage for female workers in our data. Also, we do not find a difference in pay satisfaction due to gender of the worker (nor due to the gender of the manager). Overall, as well as for very low and very high wages, females and males are equally (un)satisfied with their pay. Satisfaction with pay is elicited using a 7-point Likert-scale.

We measure working times for completion of the task as a control for possible gender differences in performance. Due to technical and procedural limitations, we only have data on a subset of workers. In this subset of 156 workers, we do not find a significant difference in working times due to gender. On average, it takes participants about 48 minutes to complete the working task. About 5 to 10 more minutes are needed to check working results for completeness and to process payoffs. Thus, the description of the working task slightly overestimates actual working times. Yet, in our data, performance does not differ between male and female workers.

[2]Please note that there is also experimental research pointing into the direction, that cooperativeness is lower towards others of the same gender (compare e.g. Ben-Ner et al. (2004); Sutter et al. (2009)).

A.4 Instructions

All participants receive the instructions on paper, managers furthermore take their pay decision on paper. The instructions consist of three parts: a welcoming sheet explaining the general rules of economic experiments (1 page), a description of the experiment, the work and a short summary (3 pages) as well as – for the manager – an answer sheet (2 pages).

We provide examples for the instructions of a manager as well as a worker that include all relevant text sections for the conducted experiment. The welcoming sheet is identical for all participants (compare figure 6). According to treatment, the instructions differ in three aspects: gender of worker or manager[3], description of the manager's trade-off and exemplification or experience of the working task. Otherwise, the instructions are held as constant as possible across treatments and as symmetric as possible for the roles of managers and workers.

The first example shows the instructions for a manager in the *ExpSelf* treatment deciding over the pay of a male worker (compare figures 7 - 9 for the description of the experiment, the working task and a summary; compare figures 10 - 11 for the respective answer sheet). The second example shows the instructions for a worker in the *Baseline* treatment in the case of a female manager (compare figures 12 - 14). Complete instructions for all other treatments and combinations are available upon request.

[3]Due to the characteristics of the German language, we used the gendered version of the word "employer" (for managers) or "employee" (for workers) in the sections after the gender has been revealed. By that, we tried to reveal gender in the most natural way possible. Overall, the gendered/female version of these words is included a total of 4 times in the instructions and once on the answer sheet for managers. The neutral/male version of the word is being used more often in the instructions.

Welcome!

Thank you for participating in an economic study at the Karlsruhe Institute of Technology (KIT).

As in all economic studies at KIT, all circumstances described in the following are true. Your decisions will be implemented exactly as described.

For participation in this study, every participant receives a show-up fee of 5 euro. In the following, you can earn additional money.

All your data and decisions will be handled confidentially and anonymously.

We would ask you to keep quiet during the study. Throughout the whole experiment, communication between the participants is not allowed.

If you have questions, please inform the experimenter by raising your hand. Your question will then be answered at your cubicle.

Figure 6: Welcome page for managers and workers. The welcome page from instructions is identical for all participants.

Instructions

In this study, there are employers and employees.

One employer decides upon the wage of one employee.

Regardless of your person, it was decided whether you are an employer or an employee.

You are an employer.

You decide upon the wage of an employee. The employee does not take **any active decision** on the wage.

As you, each employee receives 5 euro for participating in this study and additionally a payment – determined by you – for a work done. This employee is a man who also participates in this study like you.

The maximal wage for this work is 21 euro. Please determine the wage as you consider it adequate. You will receive the other part of the money in a sealed envelope at the end of this study, additionally to the 5 euro for participation. The experimenter will not know what wage you determined. Thus, you decide on the division of 21 euro, of which one part is the wage for your employee and the other part is benefitting you.

Each employee carries out the following work:

Complete disassembly of 100 pens as well as

assembly of 100 pens.

This task requires about **1 hour** of working time for each employee.

For exemplification, you will find a photo attached. On the photo, you can see an assembled pen and a completely disassembled pen. There are wo boxes with pens in front of you on the table. In one box, you will find 100 assembled pens and in one box there are 100 completely disassembled pens. All employees will work with exactly the same sorting boxes and pens.

The task will be done today or in the following days. As all employees, your employee will get to know which payment he will receive after complete execution of the work.

The employees will not know who decided upon their wage.

After you have taken your decision, we would like to ask you to anonymously and briefly answer some questions.

Page 2 of 4

Figure 7: Instructions for managers in *ExpSelf*. The example depicts the instructions for managers in *ExpSelf* deciding over a male worker (1/3).

Photo for Exemplification of the Work

An assembled pen (above)
and a completely disassembled pen (below):

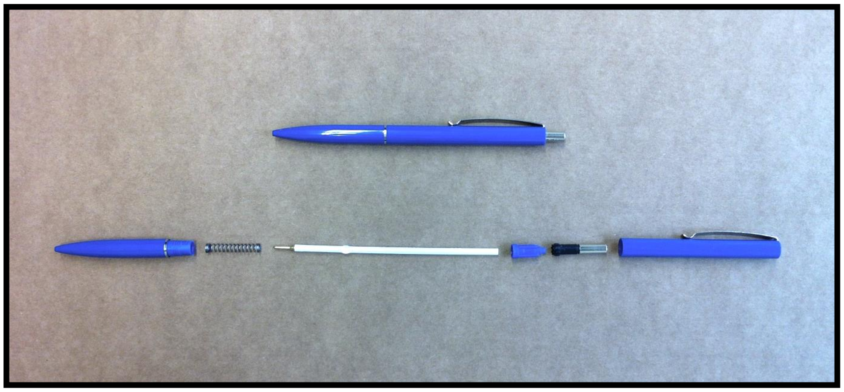

Please completely disassemble one pen and assemble one pen, now.

Please use the provided boxes for this.

Then, please continue reading the instructions.

Figure 8: Instructions for managers in *ExpSelf*. The example depicts the instructions for managers in *ExpSelf* deciding over a male worker (2/3).

Summary

Earlier and regardless of your person, it was decided that you are an employer.

You will decide upon the wage of an employee who will completely disassemble 100 pens and completely assemble 100 pens.

Your employee is a man who also participates in this study like you.

Please determine the wage how you consider it adequate. The other part of the 21 euro will benefit you. You will receive the money in a sealed envelope together with the show-up fee.

You decide with the answer sheet which lies in front of you.

Page 4 of 4

Figure 9: Instructions for managers in *ExpSelf.* The example depicts the instructions for managers in *ExpSelf* deciding over a male worker (3/3).

Answer Sheet

Please write down your Participant-ID.

Participant-ID: ______________________

Please **mark exactly one option with a cross** on the list attached, in order to communicate your decision upon the wage.

Please put the completed answer sheet back in the envelope and seal it.

Then, please inform the experimenter by raising your hand that you have finished.

As soon as all participants will have finished, the experimenter will collect the closed envelopes.

Figure 10: Answer sheet for managers in *ExpSelf*. The example depicts the answer sheet for managers in *ExpSelf* deciding over a male worker (1/2).

Please determine the wage how you consider it adequate. Please make an X in **exactly one box** in the following list:

Your decision (mark with an X)	Wage of Employee
☐	+ 0.00 €
☐	+ 0.30 €
☐	+ 0.60 €
☐	+ 0.90 €
☐	+ 1.20 €
☐	+ 1.50 €
☐	+ 1.80 €
☐	+ 2.10 €
☐	+ 2.40 €
☐	+ 2.70 €
☐	+ 3.00 €
☐	+ 3.30 €
☐	+ 3.60 €
☐	+ 3.90 €
☐	+ 4.20 €
☐	+ 4.50 €
☐	+ 4.80 €
☐	+ 5.10 €
☐	+ 5.40 €
☐	+ 5.70 €
☐	+ 6.00 €
☐	+ 6.30 €
☐	+ 6.60 €
☐	+ 6.90 €
☐	+ 7.20 €
☐	+ 7.50 €
☐	+ 7.80 €
☐	+ 8.10 €
☐	+ 8.40 €
☐	+ 8.70 €
☐	+ 9.00 €
☐	+ 9.30 €
☐	+ 9.60 €
☐	+ 9.90 €
☐	+ 10.20 €
☐	+ 10.50 €
☐	+ 10.80 €
☐	+ 11.10 €
☐	+ 11.40 €
☐	+ 11.70 €
☐	+ 12.00 €
☐	+ 12.30 €
☐	+ 12.60 €
☐	+ 12.90 €
☐	+ 13.20 €
☐	+ 13.50 €
☐	+ 13.80 €
☐	+ 14.10 €
☐	+ 14.40 €
☐	+ 14.70 €
☐	+ 15.00 €
☐	+ 15.30 €
☐	+ 15.60 €
☐	+ 15.90 €
☐	+ 16.20 €
☐	+ 16.50 €
☐	+ 16.80 €
☐	+ 17.10 €
☐	+ 17.40 €
☐	+ 17.70 €
☐	+ 18.00 €
☐	+ 18.30 €
☐	+ 18.60 €
☐	+ 18.90 €
☐	+ 19.20 €
☐	+ 19.50 €
☐	+ 19.80 €
☐	+ 20.10 €
☐	+ 20.40 €
☐	+ 20.70 €
☐	+ 21.00 €

Figure 11: Answer sheet for managers in *ExpSelf*. The example depicts the answer sheet for managers in *ExpSelf* deciding over a male worker (2/2).

Instructions

In this study, there are employers and employees.

One employer decides upon the wage of one employee.

Regardless of your person, it was decided whether you are an employer or an employee.

You are an employee.

You do work and receive a wage for that. You do not take **any active** decision on the wage.

You receive 5 euro for participating in this study as well as a wage for a work done which has been determined by your employer. Your employer is a woman who also participates in this study like you.

The maximal wage for your work is 21 euro. Your employer has determined the wage as she considered it adequate. The other part of the money will be used for other research projects which means it will be used productively. Thus, your employer decided upon the distribution of 21 euro, of which one part is your wage and the other part benefits research.

You will carry out the following work:

Complete disassembly of 100 pens as well as assembly of 100 pens.

This task requires about **1 hour** of working time for each employee.

For exemplification, you will find two photos attached. On the first photo, you can see an assembled pen and a completely disassembled pen. On the second photo you can see a box with 100 assembled pens and one box with 100 completely disassembled pens. You will work with exactly the same sorting boxes and pens.

An employer decided today or in the last days upon your wage. The amount of your wage will be disclosed and paid out in cash after the complete execution of the work.

You will not know who decided upon your wage.

Before you start your work, we would ask you to anonymously and briefly answer some questions.

Figure 12: Instructions for workers in *Baseline*. Instructions for workers in *Baseline* in the case of a female manager (1/3).

Photo for Exemplification of the Work

An assembled pen (above)
and a completely disassembled pen (below):

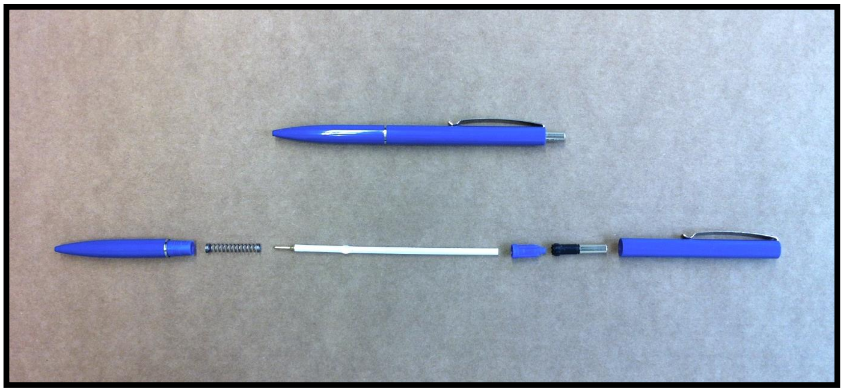

A sorting box with 100 assembled pens (left)
and a sorting box with 100 completely disassembled pens (right):

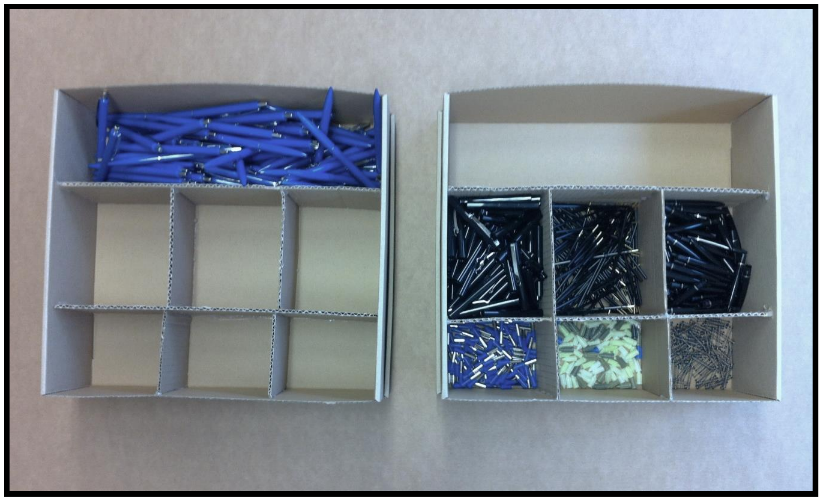

Page 3 of 4

Figure 13: Instructions for workers in *Baseline*. Instructions for workers in *Baseline* in the case of a female manager (2/3).

Summary

Earlier and regardless of your person, it was decided that you are an employee.

You will carry out the following work:

Complete disassembly of 100 pens as well as assembly of 100 pens.

An employer decided upon your wage beforehand. Your employer is a woman who participates in this study like you.

Your employer determined the wage how she considered it adequate. The other part of the 21 euro will benefit research.

You will be informed about the amount of your wage after you have completed your work and you will receive it in cash.

Page 4 of 4

Figure 14: Instructions for workers in *Baseline*. Instructions for workers in *Baseline* in the case of a female manager (3/3).

References

Allport, G. W. and H. S. Odbert (1936). Trait-Names: A Psycho-lexical Study. *Psychological Monographs 47*(1), i–171.

Auspurg, K., T. Hinz, and C. Sauer (2017). Why Should Women Get Less? Evidence on the Gender Pay Gap from Multifactorial Survey Experiments. *American Sociological Review 82*(1), 179–210.

Ben-Ner, A., F. Kong, and L. Putterman (2004). Share and Share Alike? Gender-Pairing, Personality, and Cognitive Ability as Determinants of Giving. *Journal of Economic Psychology 25*(5), 581–589.

BMAS Bundesamt für Arbeit und Soziales (2017). The Minimum Wage – Questions and answers. http://www.bmas.de/SharedDocs/Downloads/EN/PDF-Publikationen/a640-ml-broschuere-englisch.pdf?__blob=publicationFile&v=5, last accessed July 2019.

Bock, O., I. Baetge, and A. Nicklisch (2014). HROOT: Hamburg Registration and Organization Online Tool. *European Economic Review 71*, 117–120.

Charness, G. and A. Rustichini (2011). Gender Differences in Cooperation with Group Membership. *Games and Economic Behavior 72*(1), 77–85.

Christie, R. and F. L. Geis (1970). *Studies in Machiavellianism.* Academic Press.

Diekman, A. B. and E. K. Clark (2015). Beyond the Damsel in Distress: Gender Differences and Similarities in Enacting Prosocial Behavior. In *The Oxford Handbook of Prosocial Behavior*, pp. 376–391. Oxford University Press.

Eagly, A. H. (2013). *Sex Differences in Social Behavior: A Social-Role Interpretation.* Psychology Press.

Einhorn, H. J. and R. M. Hogarth (1981). Behavioral Decision Theory: Processes of Judgement and Choice. *Annual Review of Psychology 32*(1), 53–88.

Eisenberg, N. and R. Lennon (1983). Sex Differences in Empathy and Related Capacities. *Psychological Bulletin 94*(1), 100–131.

Fehr, E., L. Goette, and C. Zehnder (2009). A Behavioral Account of the Labor Market: The Role of Fairness Concerns. *Annual Review of Economics 1*, 355–384.

Fehr, E. and K. M. Schmidt (1999). A Theory of Fairness, Competition, and Cooperation. *Quarterly Journal of Economics 114*(3), 817–868.

Goetz, J. L., D. Keltner, and E. Simon-Thomas (2010). Compassion: An Evolutionary Analysis and Empirical Review. *Psychological Bulletin 136*(3), 351–374.

Greiner, B. (2015). Subject Pool Recruitment Procedures: Organizing Experiments With ORSEE. *Journal of the Economic Science Association 1*(1), 114–125.

Kahneman, D. (2003). Maps of Bounded Rationality: Psychology for Behavioral Economics. *The American Economic Review 93*(5), 1449–1475.

Rueckert, L., B. Branch, and T. Doan (2011). Are Gender Differences in Empathy Due to Differences in Emotional Reactivity? *Psychology 2*(6), 574–578.

Sawilowsky, S. S. and R. C. Blair (1992). A More Realistic Look at the Robustness and Type II Error Properties of the t Test to Departures From Population Normality. *Psychological Bulletin 111*(2), 352–360.

Scarpello, V. and S. M. Carraher (2008). Are Pay Satisfaction and Pay Fairness the Same Construct? A Cross-Country Examination Among the Self-Employed in Latvia, Germany, the UK, and the USA. *Baltic Journal of Management 3*(1), 23–39.

Schulte-Rüther, M., H. J. Markowitsch, N. J. Shah, G. R. Fink, and M. Piefke (2008). Gender Differences in Brain Networks Supporting Empathy. *Neuroimage 42*(1), 393–403.

Sutter, M., R. Bosman, M. G. Kocher, and F. van Winden (2009). Gender Pairing and Bargaining—Beware the Same Sex! *Experimental Economics 12*(3), 318–331.

Wang, L., D. Malhotra, and J. K. Murnighan (2011). Economics Education and Greed. *Academy of Management Learning & Education 10*(4), 643–660.
